# Minimum Query Set for Decision Tree Construction

**DOI:** 10.3390/e23121682

**Published:** 2021-12-14

**Authors:** Wojciech Wieczorek, Jan Kozak, Łukasz Strąk, Arkadiusz Nowakowski

**Affiliations:** 1 Department of Computer Science and Automatics, University of Bielsko-Biala, Willowa 2, 43-309 Bielsko-Biała, Poland; wwieczorek@ath.bielsko.pl; 2 Department of Machine Learning, University of Economics in Katowice, 1 Maja 50, 40-287 Katowice, Poland; 3 Faculty of Science and Technology, University of Silesia in Katowice, Bankowa 14, 40-007 Katowice, Poland; lukasz.strak@us.edu.pl (Ł.S.); arkadiusz.nowakowski@us.edu.pl (A.N.)

**Keywords:** query set, decision tree, classification

## Abstract

A new two-stage method for the construction of a decision tree is developed. The first stage is based on the definition of a minimum query set, which is the smallest set of attribute-value pairs for which any two objects can be distinguished. To obtain this set, an appropriate linear programming model is proposed. The queries from this set are building blocks of the second stage in which we try to find an optimal decision tree using a genetic algorithm. In a series of experiments, we show that for some databases, our approach should be considered as an alternative method to classical ones (CART, C4.5) and other heuristic approaches in terms of classification quality.

## 1. Introduction

One of the main problems in machine learning is finding associations in empirical data in order to optimize certain quality measures. These associations may take different forms, such as Bayesian classifiers, artificial neural networks, rule sets, nearest-neighbor or decision tree classifiers [1]. Classical decision tree learning is performed using statistical methods. However, due to the large space of possible solutions and the graph representation of decision trees, stochastic methods can also be used.

Decision trees have been the subject of scientific research for many years [2]. The most recognized algorithms in that class are ID3 [3], C4.5 [4], and CART [5]. There are also works on the evolutionary approach to generating trees. The most popular ideas connected with this research direction are described in the article of Barros et al. [6]. Other approaches, for instance, the ant colony system, also have been studied [7]. To evaluate the performance of our approach, the following methods are selected for comparison: C4.5, CART (classification and regression trees), EVO-Tree (evolutionary algorithm for decision tree induction) [8], and ACDT (ant colony decision trees) [9]. We test the predictive performance of our method using publicly available UCI data sets.

The present proposal is about the building of decision trees which maximize the quality of classification measures, such as accuracy, precision, recall and F1-score, on a given data set. To this end, we introduce the notion of minimum query sets and provide a tree construction algorithm based on that concept. The purpose of the present proposal is fourfold:

1. Defining an integer linear programming model for the minimum query set problem. It entails preparing zero-one variables along with the set of linear inequalities and an objective function before starting the searching process.
2. Devising an algorithm for the construction of a decision tree with respect to the minimum query set. The second objective is also to implement this model through an available MIP (mixed integer programming) solver to get our approach working.
3. Performing experimental studies confirming the high classification quality of the proposed method. The third objective is also to investigate to what extent the power of MIP solvers makes it possible to tackle the tree induction problem for large-size instances and to compare our approach with existing ones.
4. Sharing our program because of the possibility of future comparisons with other methods. The Crystal language implementation of our method is publicly available via GitHub. (https://github.com/w-wieczorek/mining, accessed on 8 December 2021).

This paper is organized into six sections. In Section 2, we present the necessary definitions and facts originated from the data structures and classification. Section 3 briefly introduces the related algorithms, while Section 4 describes our tree-construction algorithm based on solving an LP (linear programming) model and the genetic algorithm. Section 5 shows the experimental results of our approach with suitable statistical tests. Concluding comments and future plans are made in Section 6.

## 2. Preliminaries

In this section, we describe some definitions and facts about binary trees, decision trees, and the classification problem that are required for good understanding of our proposal. For further details about the topic, the reader is referred to the book by Japkowicz and Shah [10].

### 2.1. Observations and the Classification Problem

In supervised classification, we are given a training set called samples. This set consists of *n observations* (also called *objects*):

(1) $$X=\{x_{1},x_{2},\ldots ,x_{n}\}.$$

For each 1≤i≤n, an observation $x_{i}$ is described by *m attributes* (also called *features*):

(2) $$d\left(x_{i}\right)\in A_{1}\times A_{2}\times \cdots \times A_{m},$$

where $A_{j}$ (1≤j≤m) denotes the domain of the *j*-th attribute and $d:X\to A_{1}\times \cdots \times A_{m}$ is a function. The values of the attributes can be quantitative (e.g., a salary) or categorical (e.g., sex—“female” or “male”). Furthermore, each observation belongs to one of k≥2 different *decision classes* defined by a function c:X→C:

(3) $$c\left(x_{i}\right)\in C=\left\{c_{1},c_{2},\ldots ,c_{k}\right\}.$$

We assume that there are no two objects with the same description and different decision classes, that is, for any 1≤q, r≤n, q≠r,

(4) $$d\left(x_{q}\right)=d\left(x_{r}\right)\Rightarrow c\left(x_{q}\right)=c\left(x_{r}\right).$$

Based on the definitions given above, the *classification problem* can be defined as follows: assign an unseen object *x* to a class, knowing that there are *k* different decision classes $C=\{c_{1},c_{2},\ldots ,c_{k}\}$, each object belongs to one of them, and that $d\left(x\right)=\left(a_{1},a_{2},\ldots ,a_{m}\right)$. When k=2, we are faced with the problem called *binary classification*. A learning algorithm L is first trained on a set of pre-classified samples *S*. In practice, a set *S* consists of independently obtained samples, according to a fixed—but unknown—probability distribution. The goal of an algorithm L is to produce a “classifier” which can be used to predict the value of the class variable for a new instance and to evaluate the classification performed on some test set *V*. Thus, we can say that in the learning process, a hypothesis *h* is proposed and its classification quality can be measured by means of accuracy, precision, recall, etc.

### 2.2. Decision Trees

We define a *binary tree* recursively as a tuple (S,L,R), where *L* and *R* are binary trees or the empty set, and *S* is a singleton set containing the value of the *root*. If *L* and *R* are empty sets, *S* is called a *leaf node* (or *leaf*); otherwise, *S* is called a *non-leaf node*. If $\left(U,L_{1},R_{1}\right)$ is a binary tree and $L_{1}=\left(V_{L},L_{2},R_{2}\right)$ or $R_{1}=\left(V_{R},L_{2},R_{2}\right)$, then we say that there is an *edge* from *U* to $V_{L}$ (or from *U* to $V_{R}$). Furthermore, $V_{L}$ and $V_{R}$ are called, respectively, left and right sons of *U*.

Let $Q=\{Q_{1},Q_{2},\ldots ,Q_{t}\}$ be a collection of binary test (called *queries*) $Q_{i}:X\to \left\{0,1\right\}$, where *X* is a set of objects for which we define functions *d* and *c* as described in (2)–(4). A *decision tree*, $T_{X}$, is a binary tree in which each non-leaf node is labeled by a test from *Q* and has non-empty left and non-empty right subtrees; each leaf is labeled by a decision class; the edge from a non-leaf node to its left son is labeled 0 and the one to its right son is labeled 1. If $Q_{i_{1}},\;O_{i_{1}},\;Q_{i_{2}},\;O_{i_{2}},\ldots ,Q_{i_{h}},\;O_{i_{h}}$ is the sequence of node and edge labels on the path from the root to a leaf labeled by $c^{*}\in C$, then $c\left(x\right)=c^{*}$ for all objects x∈X for which $Q_{i_{j}}\left(x\right)=O_{i_{j}}$ for all *j* (1≤j≤h). We also require that in this manner all leaves in a decision tree cover the whole set *X*, i.e., for all x∈X, there is at least one path from the root to a leaf corresponding to *x*.

**Figure 1:**
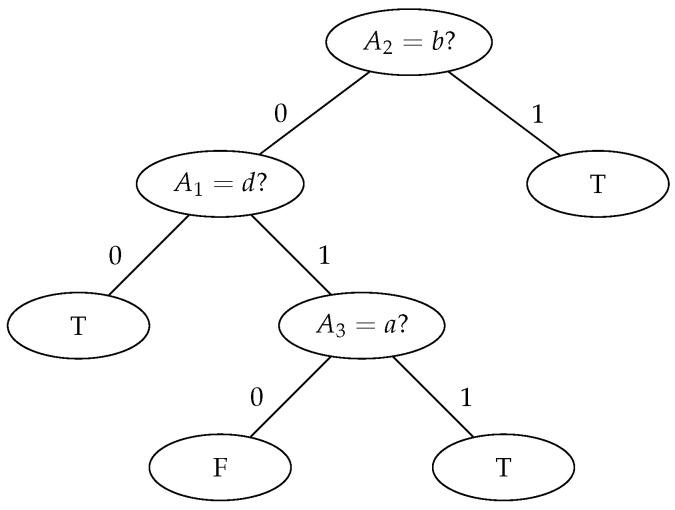
An exemplary decision tree.

The tree in Figure 1 is said to have a depth of 3. The *depth* (or *height*) of a tree is defined as the number of queries that have to be resolved down the longest path through the tree.

Naturally, every decision tree *T* can play the role of a classifier as long as the queries can be resolved for other objects, i.e., those outside the training set. Having given a new object, let us say *y*, one may apply queries from the tree starting from the root and ending in a leaf *ℓ* that points out the predicted class *p* to which the object should belong. Every query in the tree directs us to a left or right son, toward a leaf *ℓ*. We denote such a prediction as T(y)=p.

### 2.3. Quality of Classification

To assess the quality of classification, we use the classical measures of classification quality: accuracy (5), precision (6), recall (7), and F1-score (8). Notably, these are binary classification measures, i.e., for a data set with only two decision classes. However, there are often more decision classes in data sets, so we use the so-called macro method to determine the values of these measures. Thus, in the definitions, we denote the following: $TP_{i}$ to identify all correctly classified cases of the $c_{i}$ class; $TN_{i}$ to identify all cases outside the $c_{i}$ class that are not assigned to this class; $FP_{i}$ to identify all cases outside the $c_{i}$ class that are assigned to this class; $FN_{i}$ to identify all misclassified cases of the $c_{i}$ class; and *k* as the number of decision classes.

(5) $$\begin{array}{cc} acc & =\frac{1}{k}\sum_{i=1}^{k}\frac{TP_{i}+TN_{i}}{TP_{i}+TN_{i}+FP_{i}+FN_{i}} \end{array}$$

(6) $$\begin{array}{cc} pre & =\frac{1}{k}\sum_{i=1}^{k}\frac{TP_{i}}{TP_{i}+FP_{i}} \end{array}$$

(7) $$\begin{array}{cc} rec & =\frac{1}{k}\sum_{i=1}^{k}\frac{TP_{i}}{TP_{i}+FN_{i}} \end{array}$$

(8) $$\begin{array}{cc} f1 & =\frac{1}{k}\sum_{i=1}^{k}\frac{2\cdot TP_{i}}{2\cdot TP_{i}+FP_{i}+FN_{i}} \end{array}$$

## 3. Related Works

This section describes the tree construction methods taken for our comparison. These are well-known, deterministic C4.5 and CART, and stochastic, population-based algorithms: EVO-Tree and ACDT.

### 3.1. C4.5

Developed initially by Ross Quinlan in 1993 [4], the C4.5 algorithm became one of the most popular decision tree-based algorithms [11] implemented as the standard in data mining tools, i.e., Weka (https://www.cs.waikato.ac.nz/~ml/weka/, accessed on 8 December 2021). Conceptually, the heuristic is a more advanced version of the ID3 algorithm proposed by the same author in 1986 [3]. The tree-building process recursively chooses the attribute with the highest information gain ratio. The higher the information gain the attribute has, the higher position in the tree from the root it has. Each selected feature splits a node’s set of samples into subsets enriched in one class or the other [12]. To avoid over-fitting, the pruning technique is used to remove parts of the tree that minimally affect the estimated classification error. In contrast to ID3, some improvements can be made to handle missing values and continuous data [12].

### 3.2. CART

The classification and regression trees algorithm was co-authored by Leo Breiman, Jerome Friedman, Richard Olshen, and Charles Stone in 1984 [5], and is one of the most widely used decision tree making algorithms [11]. The CART is a binary (each node has two branches), recursive and non-parametric algorithm. It can be used for regression and classification problems. The decision tree making process uses the Gini impurity measure to determine attribute order in the tree [12]. The measure can be interpreted as the probability of incorrect classifying a randomly chosen observation from sample data if the attribute for the calculation is selected as the new decision tree node. The pruning mechanism is complex and produces a sequence of nested pruned trees, all candidate optimal trees. The best one is identified by evaluating the predictive performance of every tree in the pruning sequence by cross-validation.

### 3.3. EVO-Tree

The EVO-Tree algorithm [8] is an evolutionary algorithm that generates binary decision trees for classification. It uses the minimization of a multi-objective fitness function that utilizes the balance between the number of correctly classified instances and the size of the generated decision tree. The algorithm starts with the randomly initialized population of trees and uses two standard genetic operators: crossover and mutation. The crossover creates offspring by replacing a randomly selected sub-tree in the first parent with a sub-tree from the second parent. The parents’ selection is made in a series of tournaments. In each tournament, a certain number of individuals from the population is randomly picked. Then, the best individual in terms of the fitness function value is chosen as a tournament winner to be put into the the pool of parents. The mutation randomly changes both attribute and split value of a decision tree. Finally, the algorithm stops if the maximum number of generations is reached or the fitness of the best individual does not improve after a fixed number of iterations.

### 3.4. ACDT

The ant colony decision tree (ACDT) algorithm [7] is an application of ant colony optimization algorithms [13] in the process of constructing decision trees. The good results typically achieved by the ant colony optimization algorithms when dealing with combinatorial optimization problems suggest the possibility of using that approach for the efficient construction of decision trees [14,15]. In the ACDT algorithm, each agent ant chooses an appropriate attribute for splitting the objects in each node of the constructed decision tree according to the heuristic function and pheromone values. The heuristic function is based on the twoing criterion (known from the CART algorithm) [5,16], which helps agent ants divide the objects into two groups. In this way, the attribute which best separates the objects is treated as the best condition for the analyzed node. Pheromone values represent the best way (connection) from the superior to the subordinate nodes—all possible combinations in the analyzed subtrees. For each node, the following values are calculated according to the objects classified, using the twoing criterion of the superior node.

## 4. Proposed Method

Our learning algorithm L receives as its input samples *S*, which are split into two subsets, the training set *X* and the test set *Y* (in experiments, we chose the proportions 4/7 to *X* and 3/7 to *Y*). Hypothesis space $H_{L}={\left\{T_{X}^{i}\right\}}_{i\in I}$ is searched in order to find a decision tree that approximates best the unknown true function. To this end, each tree is validated against *Y*: as a result, we output a tree $T_{X}^{*}$ that minimizes $\mathrm{err}=|\{y\in Y:T_{X}^{*}\left(y\right)\neq c\left(y\right)\}|$. Unfortunately, in practice, we are not able to cover the whole hypothesis space. The selected hypothesis $T_{X}^{*}$ can then be used to predict the class of unseen examples in the validation set, taken for the evaluation of L. More exactly, L has two stages. In the first stage, by means of zero-one linear programming, a minimum query set *Q* is determined. In the second stage, by means of the genetic algorithm, the best ordering of *Q*—in the view of a decision tree construction—is settled. Let x∈X, $d\left(x\right)=\left(a_{1},a_{2},\ldots ,a_{m}\right)$, and $v\in A_{j}$ (1≤j≤m). In our approach, a *query* can be a function defined by $Q_{i}\left(x\right)=1$ if $a_{j}=v$ and $Q_{i}\left(x\right)=0$ if $a_{j}\neq v$. Thus, non-leaf nodes contain “questions” such as $A_{j}=v?$.

We require *Q* to be a minimum size query set satisfying the following condition: for each pair of distinct elements u,w∈X with c(u)≠c(w), there is some query q∈Q that q(u)≠q(w). We verified experimentally that this minimality is crucial in achieving good quality decision trees.

### 4.1. Linear Program for the Minimum Query Set Problem

Let us show how a collection of queries, *Q*, is determined via an integer program for the training set $X=\{x_{1},x_{2},\ldots ,x_{n}\}$. The integer variables are $z_{jv}\in \left\{0,1\right\}$, 1≤j≤m, $v\in A_{j}$, assuming that there are *m* attributes, $A_{1},\;A_{2},\ldots ,A_{m}$. The value of $z_{jv}$ is 1 if some query in *Q* is defined with $A_{j}$ and $v\in A_{j}$; in other words, $A_{j}=v?$ is taken as a non-leaf node label representing the query and $z_{jv}=0$ otherwise, i.e., there is no query based on $A_{j}$ and *v*. Let us now see how to describe the constraints of the relationship between a set *Q* and a set *X*, with features and classes defined by functions *d* (as in (2)) and *c* (as in (3)), in terms of linear inequalities. For every pair of distinct elements u,w∈X with c(u)≠c(w), we should have at least one query that distinguishes between the two. The following equation is the standard way of showing in a linear program that some elements (i.e., queries modeled as 0–1 variables) have to be included in the solution:

(9) $$\sum_{\begin{array}{c} 1\leq j\leq m\\ a_{j}\neq b_{j} \end{array}}z_{ja_{j}}+z_{jb_{j}}\geq 1,$$

where $\left(a_{1},a_{2},\ldots ,a_{m}\right)=d\left(u\right)$ and $\left(b_{1},b_{2},\ldots ,b_{m}\right)=d\left(w\right)$. Obviously, we are to find the minimum value of the linear expression

(10) $$\sum_{\left\{\left(j,v\right):\;1\leq j\leq m,\;v\in A_{j}\right\}}z_{jv}.$$

Please note that the above-mentioned problem is computationally complex (that is why we use an LP solver, specifically Gurobi optimizer) since Garey and Johnson’s [17] NP-complete problem SP6 can be easily transformed to the decision version of the minimum query test problem.

### 4.2. The Construction of a Decision Tree with the Help of the Genetic Algorithm

After obtaining a minimum query set $Q=\{Q_{1},Q_{2},\ldots ,Q_{t}\}$, we are ready to create a decision tree $T_{X}$ by Algorithm 1.
**Algorithm 1** A recursive algorithm for the construction of $T_{X}$. **function** BuildTree(X,Q)            ▹ objects *X* as set, queries *Q* as array     **if** all x∈X have the same decision c(x) **then**           **return** ({c(x)},∅,∅)                      ▹ a leaf inside     **else**           find first *i* for which $Q_{i}$ splits *X* into to non-empty sets           $X_{L}\;=\;\left\{x\in X:Q_{i}\left(x\right)=0\right\}$           $X_{R}\;=\;\left\{x\in X:Q_{i}\left(x\right)=1\right\}$           **return** $(\left\{Q_{i}\right\},$ BuildTree($X_{L},Q$), BuildTree($X_{R},Q$))     **end if** **end function**

**Theorem** **1.** *Let X be a set of n ≥ 1 observations and let $Q\;=\;\{Q_{1},\ldots ,Q_{t}\}$ be a set of such queries that for every pair of distinct elements u,w∈X with c(u)≠c(w) there is some i (1≤i≤t) for which $Q_{i}\left(u\right)\neq Q_{i}\left(w\right)$. Then* BuildTree *(X,Q) constructs a decision tree for X.*

**Proof.**  Let $T_{X}$ be a tree returned by BuildTree(X,Q). The conclusion of the theorem can be written as follows: $T_{X}\left(x\right)=c\left(x\right)$ for an arbitrary x∈X. We prove it by induction on *n*.

Basis: We use n=1 as the basis. The tree consisting of one leaf is returned, with the decision c(x), so $T_{X}\left(x\right)=c\left(x\right)$, where *x* is the only element of *X*.

Induction: Suppose that the statement of the theorem holds for all k<n, where k=|X|. We want to show that for an arbitrary x∈X, where |X|=n, $T_{X}\left(x\right)=c\left(x\right)$ holds. Let us consider two cases: (i) all x∈X have the same decision c(x), and (ii) there is such y∈X that c(x)≠c(y). In the former case, we can easily verify that $T_{X}\left(x\right)=c\left(x\right)$. In the latter case, there is some *i* (1≤i≤t) for which $Q_{i}$ splits *X* into two non-empty sets, $X_{L}$ and $X_{R}$. An element *x* is put into one of them. If it is $X_{L}$ (i.e., $x\in X_{L}$), by the inductive hypothesis, we can claim that $T_{X_{L}}\left(x\right)=c\left(x\right)$, where $T_{X_{L}}$ is the left subtree of a non-leaf node containing $Q_{i}$. Thus, $T_{X}\left(x\right)=c\left(x\right)$. For $x\in X_{R}$, we can repeat our reasoning.

Therefore, by strong induction, BuildTree(X,Q) constructs a decision tree for any set *X* of n≥1 observations. □

Please notice that the shape of a tree $T_{X}$ depends on the ordering of queries in an array *Q*. As a consequence, the order decides the quality of classification done by a tree returned by function BuildTree. That is why we apply the genetic algorithm (Algorithm 2) as a heuristic method to search such a large solution space [18]. Each individual is the permutation of the set {1,2,…,t}, which determines the order of $Q=\{Q_{1},Q_{2},\ldots ,Q_{t}\}$.
**Algorithm 2** The genetic algorithm for finding an optimal permutation. **function** GeneticAlgorithm     make an initial population *P* of POP_SIZE individuals     iteration :=0     **while** iteration < MAX_ITER and err(best_ind) > 0 **do**         iteration := iteration + 1         select T_SIZE elements from *P*         recombine two best of them by means of PMX         replace the worst selected element with the child         mutate it with a probability PROB_MUTATION     **end while**     **return** best_ind **end function**

The population size depends on the complexity of the problem, but usually contains several hundreds or thousands of possible solutions. We follow the advice of Chen et al. [19] and take POP_SIZE=2tlnt (they suggested |P|=O(lnn), where *n* is the problem size, while our *n* is t!). The initial population is generated randomly, allowing the entire range of possible permutations.

During each successive iteration, a portion of the existing population (T_SIZE=3 is chosen during preliminary experiments) is selected to breed a new individual. Solutions are selected through a fitness-based process, where fitter solutions (as measured by a fitness function) are chosen to be parents.

The fitness function is defined over the genetic representation and measures the quality of the represented solution. We use Algorithm 1 to decode a permutation. The number of misclassified objects for a test set *Y* is the fitness value.

For each new solution to be produced, a pair of “parent” solutions is selected for breeding from the pool selected previously. By producing a “child” solution using the crossover and mutation operations, a new solution is created which typically shares many of the characteristics of its “parents”. We use partially mapped crossover (PMX for short) because it is the most recommended method for sequential ordering problems [18,20]. In the mutation operation, two randomly selected elements of a permutation are swapped with a probability PROB_MUTATION=0.01. This process is repeated until one of the two termination condition is reached: (i) a solution is found that satisfies minimum criteria, or (ii) fixed number (MAX_ITER=500t) of iterations reached. As a result, the best permutation encountered during all iterations is returned.

The final Algorithm 3 is depicted below. Note that heuristic search procedures that aspire to find globally optimal solutions to hard optimization problems usually require some diversification to overcome the local optimality. One way to achieve diversification is to restart the procedure many times [21]. We follow this advice and call the genetic algorithm 30 times, returning the best solution found over all starts.
**Algorithm 3** The final algorithm.**Require:** S=X∪Y the set of objects with functions *d* and *c***Ensure:** a decision tree $T_{X}$ that tries to match a subset *Y* define a linear programming model according to (9) and (10) solve the model to obtain a minimum query set $Q=\{Q_{1},\ldots ,Q_{t}\}$ multiple times run GeneticAlgorithm to obtain a permutation π **return** BuildTree($X,\;[Q_{\pi (1)},Q_{\pi (2)},\ldots ,Q_{\pi (t)}]$)


Because our algorithm relies on solving the minimum query set problem (finding the minimum set of attribute-value pairs that distinguishes every two objects) that is NP-hard, its overall complexity is exponential with respect to the size of input data. To tackle the problem, we use an integer linear programming solver. As modern ILP solvers are very ingenious, for practical data sets the computing time is not a big problem. Algorithms for solving ILP-problems and their NP-completeness were described in the book of [22].

## 5. Experiments

The section describes the comparison between selected referenced methods introduced in Section 3 and our proposed Algorithm 3 devised in the previous section.

### 5.1. Benchmark Data Sets

To verify our approach, we select 11 publicly available data sets with different numbers of objects, attributes, and decision classes. Used data sets are downloaded from the UCI data sets repository (https://archive.ics.uci.edu/, accessed on 8 December 2021) and are not subject to any modifications, except for possible ID removal. They are presented in Table 1, where the abbreviation used further in the paper is given in brackets, followed by the number of objects in the data set, the number of attributes, and the number of decision classes.

### 5.2. Performance Comparison

In this section, we describe some experiments comparing the performance of our approach implemented (https://github.com/w-wieczorek/mining, accessed on 8 December 2021) in Crystal language with ACDT implemented (https://github.com/jankozak/acdt_cpp, accessed on 8 December 2021) in C++, Weka’s C4.5 implemented in Java, Scikit-learn’s CART and EVO-Tree implemented (https://github.com/lazarow/dtree-experiments, accessed on 8 December 2021) in Python.

For the purpose of the experimental study, all data sets described in Section 5.1 are divided into three sets: training set (40%), test set (30%), and validation set (30%). For the classical algorithms (CART, C4.5) and EVO-Tree, the training and test sets are combined and used to learn the algorithm, while for the other algorithms, the training and test sets are used separately (according to the rule of the algorithm). In each case, the results are verified through the validation set. In this section, all given values are the results of classification performed on the validation set. So a train-and-test approach is used, but it is ensured that the data breakdowns are exactly the same in each case.

**Figure 2:**
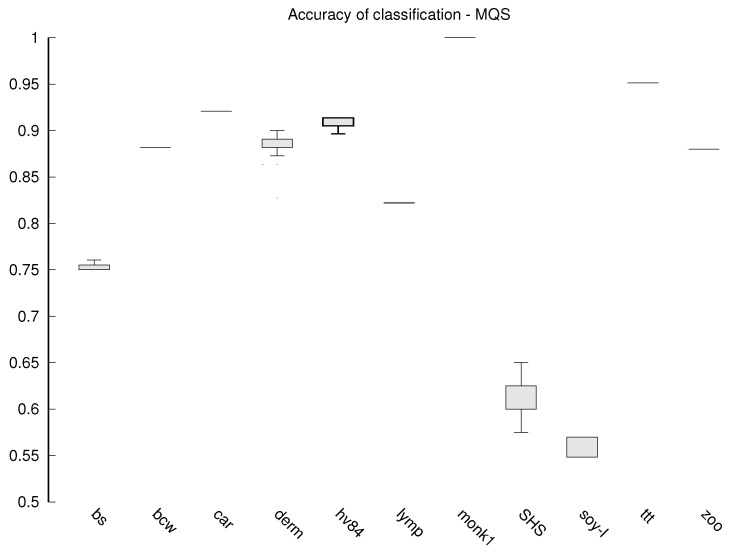
Box plot—accuracy of classification for the MQS algorithm.

**Figure 3:**
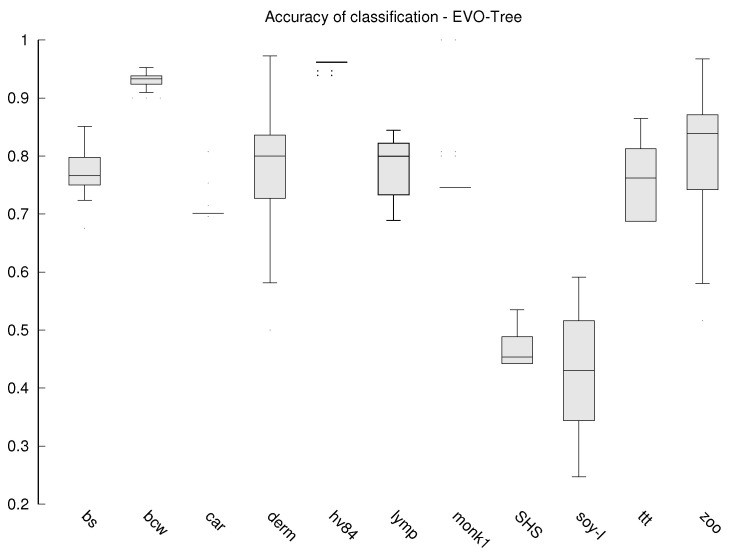
Box plot—accuracy of classification for the EVO-Tree algorithm.

**Figure 4:**
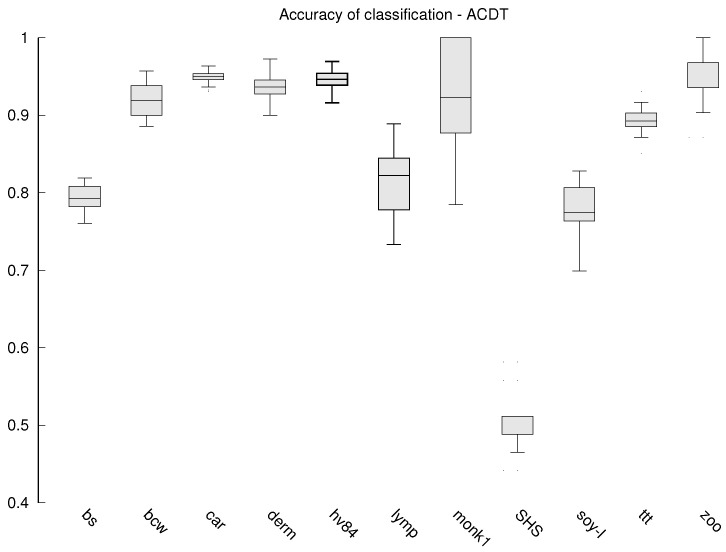
Box plot—accuracy of classification for the ACDT algorithm.

Additionally, for the algorithms that do not work deterministically (the proposed MQS and the compared EVO and ACDT) each experiment is repeated 30 times and the values presented in Table 2 and Table 3 are the averages. The stability of the results obtained by these algorithms is also tested, which is presented in the form of box plots in Figure 2, Figure 3 and Figure 4.

### 5.3. Results of Experiments

The proposed algorithm is compared with two classical approaches and two heuristic algorithms (another genetic algorithm and the ant colony optimization algorithm). Our goal was to experimentally verify whether the MQS algorithm allows finding different (often better) solutions than the compared algorithms. The achieved results show that our assumption is confirmed.

The MQS algorithm, in terms of the analyzed metrics (see Section 2.3), allows for a significant improvement in the results for 3 out of 11 data sets. Thus, in the case of the monks-1 data set, the improvements in classification quality of almost 5% (with respect to CART), almost 7% (with respect to ACDT), about 16% (with respect to C4.5), and as much as about 20% with respect to another genetic algorithm (EVO-Tree) are obtained. There is an even greater improvement for the 2015 Somerville Happiness Survey data set and slightly less for tic-tac-toe.

For the remaining data sets, the MQS algorithm obtains similar or slightly worse results, but only in one case the difference in classification quality is large—this is for the soybean-large data set. However, in two more cases, it is noticeable: dermatology and zoo. In each of these cases, the second GA algorithm has also poorer classification quality. As can be seen, the problem concerns sets with a large number of attributes (34 for dermatology, 16 for soybean-large, and 16 for zoo), so as the solution space increases (for classification, it depends on the number of attributes and the values of these attributes), the MQS algorithm has a harder time finding a suitable solution.

Our aim is to propose a new algorithm that will allow finding new optima in the solution space (in terms of classification quality). Thus, in some cases, it will allow to improve the quality of classification compared to other algorithms. Therefore, we do not try to improve either the size of the tree, the height of the tree, or the algorithm’s running time, which is hard to compare between genetic and deterministic algorithms. However, we make a comparison of these decision tree-related parameters, and the results are shown in Table 3.

As can be seen, the MQS algorithm is similar in the decision tree learning time to another algorithm related to genetic algorithms (EVO-Tree). However, in terms of decision tree size and height, the proposed algorithm mostly constructs the largest trees. This is probably related to searching the solution space and covering the solution with the local optima. The size of the decision tree does not correlate with its classification quality (in relation to other algorithms) and so a significantly larger tree, e.g., in the case of the balance-scale data set, does not improve the results, while in the case of tic-tac-toe, the results are improved while increasing the decision tree.

The stability of the results obtained is also subject to our analysis, because the stability allows us to assume that the classifier will always be of similar quality. While in the case of classical algorithms, the results are deterministic, in the case of MQS, EVO-Tree and ACDT, a different classifier may emerge each time. Box plots are prepared with classification accuracy for each data set in case of MQS (Figure 2), EVO-Tree (Figure 3), and ACDT (Figure 4) algorithms. To prepare the graphs, the corresponding quantiles (minimum value is lowest on the OY axis, 1st quantile, 2nd quantile (median), 3rd quantile and maximum value that is highest on the OY axis) from all 30 repetitions of learning the decision tree are determined.

The MQS algorithm is the most stable; in Figure 2, we can see that only for the Somerville Happiness Survey 2015 and soybean-large data set, small (compared to the other algorithms) differences appear. For the other data sets, the results are very repeatable. For the other algorithms, the repeatability of the results is much lower, and so for EVO-Tree, we can see in Figure 3 that in seven cases, the differences are quite divergent; for the dermatology, soybean-large and tic-tac-toe databases, the classification accuracy in successive repetitions changes even by several dozen percentage points. In the case of the ACDT algorithm, the results are more reproducible (Figure 4)—significant differences appear in two to three cases, while for the monks-1 set, the difference can be as much as several dozen percentage points.

### 5.4. Statistical Analysis

The experimental results of the MQS approach are compared using a non-parametric statistical hypothesis test, i.e., the Friedman test [23,24] for α=0.05. Parameters of the Friedman test are shown in Table 4. The same table presents the average rank values for the compared algorithms for learning decision trees (in terms of classification quality). Results in terms of each of the classification quality measures analyzed are used for statistical testing.

The MQS algorithm obtains a rank of 3.1591, so it is significantly better than the EVO-Tree algorithm (the 5% critical difference is 0.6192); MQS is worse than the other algorithms, but this is by no means a critical difference. Therefore, we confirm that it is possible to use the MQS algorithm in the decision tree learning process, so it should always be considered and tested because it can output a significantly better classifier than the other algorithms. This is especially valid when we are given a data set with a small number of attributes. At the same time, we confirm that the proposed algorithm is significantly better than another genetic algorithm used for decision tree learning.

As the EVO-Tree algorithm is found to be critically inferior to all other approaches analyzed, we perform a second round of statistical analysis. The results of the Friedman test and the mean ranks after rejecting the critically inferior method are recorded in Table 5. As can be seen, in this case, none of the methods is critically better or worse than all the others. The big difference remains only when contrasting MQS with CART.

Due to the lack of significant differences and the advantage of obtaining significantly higher results (when the MQS algorithm gets a rank of 1, it is better by several/dozen percentage points, where in other methods, the advantage is often negligible—see Table 2), the proposed method can be considered for use in selected classification problems.

### 5.5. Discussion

To evaluate the proposed algorithm, we made comparisons with classical approaches and other non-deterministic algorithms. This is a new algorithm proposal, so we wanted to make a fair comparison. We used up to four different measures of classification quality. We also compared the size and height of the decision tree and the learning time of the classifier. Finally, we performed statistical tests.

As decision trees learned with non-deterministic methods often search a much larger solution space, this must affect their running time. It can also result in larger, more extensive decision trees. When proposing the MQS algorithm, we knew that the classifier learning time would require more time. Therefore, its application, like other stochastic methods, should be considered for classifiers that are built once in a while—not online classifiers. Our study confirmed that the MQS algorithm takes longer to learn than statistical methods. However, it is comparable to non-deterministic methods (especially another genetic algorithm).

In this case, the classification time is more important, and it depends primarily on the height of the decision tree. Our analyses indicated, for example, that the MQS algorithm is better than the CART algorithm in 10 out of 11 cases, remaining worse than the other algorithms in 7–9 cases. In terms of the size of the decision trees (this affects the memory occupation needed to store the finished classifier), the situation is similar. The MQS and CART algorithm learn larger decision trees than the others. However, it should be emphasized that no pruning of decision trees is performed for the proposed MQS algorithm. At this stage, we wanted to keep the complete decision trees.

However, our aim was to find new alternative classifiers with which a better classification could be achieved. Therefore, the most important analysis concerned the evaluation of classification quality. In this case, we were able to see that for some data sets, the MQS algorithm allows to build a classifier better than all other algorithms.

This is particularly important because often the differences (in classification quality assessment) between different algorithms are a few percentage points. However, for the monks-1, Somerville Happiness Survey 2015 and tic-tac-toe data sets, the MQS algorithm allows a very large improvement in each of the classification quality assessment measures.

We analyzed the exact structure of these data sets. Our observations show that the application of the proposed algorithm can be particularly beneficial for data sets with two decision classes and attributes with a small number of possible values (3–5 values of each attribute). However, the decision classes can be of different numbers. This does not mean, however, that the MQS algorithm obtains bad results with other sets—the suggestion described above indicates a situation where a classifier learned by MQL obtains results with much better classification quality.

Finally, we analyzed the stability of the results obtained. We did this to determine whether the classifiers learned by the MQS algorithm are always of similar quality. For this purpose, we performed 30 independent runs of the algorithm and obtained 30 independent classifiers. We performed the same tests with other stochastic algorithms (EVO-Tree and ACDT). The obtained results clearly indicate that the proposed algorithm is the most stable one, so it can be assumed that the classifier will always obtain similar results.

To confirm our observations, a statistical test was performed twice: the first time, for all approaches (and all classification quality values) and the second time, after rejecting the EVO-Tree algorithm (it obtained results with a critical difference with respect to other algorithms). This time, the critical difference of one algorithm against all others was not shown.

## 6. Conclusions

This paper deals with the construction of decision trees based on the finite set of observations (objects). In order to address the problem, we introduced the notion of minimum query set and made use of the genetic algorithm for suitable ordering of the found queries. As the result of the implemented algorithm, we achieved decision trees that perfectly match the training data set and have good classification quality on the test set. The conducted experiments and statistical inference showed that the new proposed, two-stage algorithm should be considered as an alternative method to classical ones (CART, C4.5) and other heuristic approaches in terms of accuracy, precision, recall, and F1-score for all 11 UCI data sets.

Our method has also a few disadvantages. The most significant ones are that (i) the first stage of our approach relies on solving a computationally intractable problem, and (ii) for some cases, the obtained decision trees have too many nodes. In the near future, we are planning to adapt our approach to handle continuous attributes. In order to make it possible to reproduce our results or apply our method on new data, we share the source code of all algorithms via the Github platform.

## Figures and Tables

**Table 1:** Characteristics of data sets.

Data Set	Objects	Number of Attributes	Classes
balance-scale (bs)	625	4	3
breast-cancer-wisconsin (bcw)	699	9	2
car (car)	1728	6	4
dermatology (derm)	366	34	6
house-votes-84 (hv84)	435	16	2
lymphography (lymp)	148	18	4
monks-1 (monk1)	432	6	2
Somerville Happiness Survey 2015 (SHS)	143	6	2
soybean-large (soy-l)	307	35	19
tic-tac-toe (ttt)	958	9	2
zoo (zoo)	101	16	7

**Table 2:** The quality of classification depending on the approach (bold text is the best value).

Data Set	Measure	MQS	C4.5	CART	EVO	ACDT
bs	acc	0.7551	0.6809	**0.8085**	0.7730	0.7936
	pre	0.5559	0.4562	**0.5891**	0.5196	0.5482
	rec	0.5360	0.4843	**0.5739**	0.5505	0.5646
	f1	0.5436	0.4656	**0.5783**	0.5290	0.5538
bcw	acc	0.8817	**0.9333**	0.9190	0.9317	0.9192
	pre	0.8855	**0.9340**	0.9252	0.9270	0.9144
	rec	0.8808	0.9261	0.9059	**0.9313**	0.9173
	f1	0.8812	**0.9300**	0.9135	0.9290	0.9158
car	acc	0.9210	0.9056	**0.9730**	0.7069	0.9492
	pre	0.7946	0.7667	**0.9267**	0.3029	0.8511
	rec	0.8565	0.7600	**0.9329**	0.2609	0.9131
	f1	0.8205	0.7630	**0.9275**	0.2306	0.8714
derm	acc	0.8861	**0.9364**	0.9273	0.7879	0.9361
	pre	0.8605	**0.9334**	0.9152	0.7753	0.9276
	rec	0.8478	0.9244	0.9157	0.7225	**0.9248**
	f1	0.8488	**0.9278**	0.9142	0.7293	0.9253
hv84	acc	0.9078	0.9466	0.9313	**0.9603**	0.9450
	pre	0.8897	0.9300	0.9224	**0.9528**	0.9385
	rec	0.9096	0.9534	0.9326	**0.9641**	0.9442
	f1	0.8981	0.9436	0.9269	**0.9578**	0.9412
lymp	acc	**0.8222**	**0.8222**	**0.8222**	0.7896	0.8163
	pre	0.5411	**0.7613**	0.6677	0.6178	0.5764
	rec	0.6683	**0.9122**	0.6722	0.4980	0.5741
	f1	0.5837	**0.7912**	0.6679	0.5290	0.5718
monk1	acc	**1.0000**	0.8385	0.9538	0.7959	0.9331
	pre	**1.0000**	0.8807	0.9548	0.8469	0.9330
	rec	**1.0000**	0.8333	0.9548	0.7899	0.9330
	f1	**1.0000**	0.8323	0.9538	0.7857	0.9330
SHS	acc	**0.6125**	0.4419	0.4186	0.4682	0.4985
	pre	**0.6481**	0.5974	0.4378	0.5837	0.6118
	rec	**0.6500**	0.5428	0.4352	0.5532	0.5785
	f1	**0.6124**	0.4028	0.4173	0.4481	0.4844
soy-l	acc	0.5634	0.8478	**0.8495**	0.4706	0.7789
	pre	0.4974	**0.8565**	0.8560	0.4912	0.7173
	rec	0.6348	**0.8553**	0.8382	0.3224	0.6909
	f1	0.5294	0.8229	**0.8232**	0.3418	0.6367
ttt	acc	**0.9514**	0.8368	0.9132	0.7434	0.8927
	pre	**0.9626**	0.8092	0.8951	0.7387	0.8978
	rec	**0.9253**	0.8146	0.9066	0.6175	0.8485
	f1	**0.9412**	0.8118	0.9005	0.6217	0.8675
zoo	acc	0.8800	**0.9677**	**0.9677**	0.8720	0.9505
	pre	0.7381	**0.9524**	0.7857	0.7998	0.9080
	rec	0.8163	**0.9643**	0.8571	0.7539	0.8964
	f1	0.7636	**0.9510**	0.8095	0.7587	0.8857

**Table 3:** Decision tree characteristics depending on the approach.

Data Set	Parameter	MQS	C4.5	CART	EVO	ACDT
bs	time[s]	76.1	<0.1	<0.1	20.5	0.3
	size	257.1	31.0	241.0	15.1	79.4
	height	14.9	4.0	10.0	8.1	8.9
bcw	time[s]	11.7	<0.1	<0.1	12.5	0.2
	size	51.1	22.0	71.0	9.1	18.0
	height	8.0	3.0	12.0	5.4	5.7
car	time[s]	114.1	<0.1	<0.1	11.2	0.5
	size	318.9	134.0	163.0	1.7	109.4
	height	13.3	6.0	14.0	1.5	11.8
derm	time[s]	26.6	<0.1	<0.1	10.2	0.4
	size	64.7	25.0	27.0	10.8	16.6
	height	9.0	7.0	10.0	5.7	6.8
hv84	time[s]	2.6	<0.1	<0.1	4.3	0.1
	size	31.6	7.0	41.0	3.8	16.4
	height	5.9	3.0	6.0	2.5	4.2
lymp	time[s]	1.2	<0.1	<0.1	5.5	0.1
	size	34.0	20.0	49.0	11.0	20.0
	height	6.0	6.0	7.0	6.2	5.0
monk1	time[s]	0.1	<0.1	<0.1	3.9	0.1
	size	20.3	32.0	89.0	4.1	23.0
	height	5.0	5.0	10.0	2.6	6.1
SHS	time[s]	2.4	<0.1	<0.1	2.5	0.1
	size	64.3	9.0	87.0	8.0	15.6
	height	7.9	3.0	13.0	4.2	6.1
soy-l	time[s]	14.6	<0.1	<0.1	14.3	1.3
	size	151.8	67.0	75.0	14.0	45.8
	height	9.3	9.0	17.0	6.6	8.8
ttt	time[s]	24.9	<0.1	<0.1	17.5	0.4
	size	228.2	124.0	151.0	7.8	54.2
	height	9.0	7.0	11.0	4.1	8.0
zoo	time[s]	0.5	<0.1	<0.1	3.3	<0.1
	size	19.1	15.0	19.0	9.0	13.2
	height	4.9	6.0	7.0	5.1	4.9

**Table 4:** The Friedman test results and mean ranks.

	**Values**
N	44
Chi-Square	24.0594
degrees of freedom	4
*p* value is less than	0.0001
5% critical difference	0.6192
**Mean ranks**	
MQS	3.1591
C4.5	2.6932
CART	2.5568
EVO	3.9545
ACDT	2.6364

**Table 5:** Friedman test results and mean ranks after rejection of the critically worse method.

	**Values**
N	44
Chi-Square	5.8
degrees of freedom	3
*p* value is less than	0.1218
5% critical difference	0.5305
**Mean ranks**	
MQS	2.8864
C4.5	2.4205
CART	2.2614
ACDT	2.4318

## Data Availability

Not applicable.
